# Laying Stage-Modulated Effects of Feed Restriction on Volatile Compounds of Wenchang Chicken Muscle Assessed by E-Nose, GC-MS and GC-IMS

**DOI:** 10.3390/foods15183326

**Published:** 2026-09-19

**Authors:** Yunpeng Huang, Meiling Chen, Qicheng Jiang, Xinli Zheng, Tieshan Xu, Lihong Gu

**Affiliations:** 1 Institute of Animal Science and Veterinary Medicine, Hainan Academy of Agricultural Sciences, Haikou 571199, China; 2 Key Laboratory of Tropical Animal Breeding and Disease Research, Xia Xianzhu Academician Team Innovation Center, Haikou 571100, China; 3 Tropical Crops Genetic Resources Institute, Chinese Academy of Tropical Agricultural Sciences, Haikou 571101, China

**Keywords:** Wenchang chicken, volatile flavor compounds, feed restriction, laying stage, coconut chicken

## Abstract

Wenchang chicken is a premium indigenous breed in China, yet the combined effects of laying stage and feed restriction on its volatile meat flavor remain poorly understood. Breast and thigh muscles from Wenchang hens at 160, 180, 210, and 360 days (*n* = 3/treatment) under feed restriction (FR) or ad libitum (AL) feeding were prepared using the traditional coconut chicken cooking protocol and analyzed by electronic nose, HS-SPME-GC-MS, and HS-GC-IMS, using three-way ANOVA and CLR-transformed PLS-DA. Muscle type was the dominant main effect for meat color (L*: *p* = 0.004; a*: *p* < 0.001; b*: *p* < 0.001), while laying stage dominated key lipid-oxidation volatiles (octanal, dodecane, tetradecane; all *p* < 0.001). The age × feeding interaction was significant for seven key compounds (all *p* < 0.05), confirming stage-dependent reversal: AL increased aldehydes and alkanes at 160–180 days but reversed at 360 days. Physical traits were nonsignificant. GC-MS identified 79 reproducible compounds; PLS-DA confirmed significant discrimination by feeding (Q2Y = 0.830), age (Q2Y = 0.089), and muscle (Q2Y = 0.148). These findings demonstrate combined regulation of age, feeding, and muscle cut on flavor, supporting optimal quality windows for Wenchang chicken products.

## 1. Introduction

Poultry meat quality is increasingly evaluated by sensory and flavor attributes alongside nutritional value, driven by rising consumer demand for premium products [1,2]. Wenchang chicken, a nationally protected indigenous breed from Hainan Province, China, represents a high-value segment of the local poultry industry and serves as the core ingredient of the iconic coconut chicken hotpot. Its characteristic tender texture and delicate aroma are jointly shaped by genetic background, rearing regime, and physiological stage [3,4]. For laying hens, the progression of the laying cycle drives profound endocrine and metabolic remodeling: dramatic shifts in estrogen levels, lipid mobilization, and muscle protein turnover directly alter intramuscular fat deposition, fatty acid composition, and free amino acid pools in skeletal muscle—all of which are primary precursors of cooked meat flavor [5,6,7].

Feed restriction is a standard management practice in layer production to control body weight, improve laying performance, and reduce metabolic disorders [8,9]. However, how feed restriction interacts with laying stage to modify muscle flavor precursor accumulation remains largely unexplored in indigenous chicken breeds. Unlike fast-growing broilers, Wenchang chickens are typically marketed across a wide age range covering pre-laying to late laying stages, meaning flavor quality varies substantially along the production cycle. Defining how age and feeding regime jointly shape meat flavor would directly support precise quality control and optimal slaughter window selection for the industry [10].

Complicating this question is the unique cooking tradition of Hainan coconut chicken. In this preparation, chicken pieces are simmered in fresh coconut water with coconut flesh and minimal seasoning, so the final sensory profile depends heavily on the intrinsic flavor of the meat itself. Meanwhile, sugars, minerals, and bioactive components in the coconut matrix may participate in thermal reactions and modify volatile release during cooking [11,12,13]. To date, most flavor studies on Wenchang chicken have focused on cooking methods or breed comparison, and no study has systematically examined the combined effects of laying stage and feed restriction under coconut chicken cooking conditions.

Building on our previously validated multi-technique analytical framework combining electronic nose, HS-SPME-GC-MS, and HS-GC-IMS [14], the present study investigates breast and thigh muscles of Wenchang hens at four laying stages (160, 180, 210, and 360 days) under feed restriction or ad libitum feeding, all prepared following the traditional coconut chicken protocol. Our results demonstrate that laying age is the primary driver of flavor variation, and that the effect of feed restriction on volatile profiles is strongly stage-dependent, with a reversed pattern observed at the end-laying stage. The objective is to reveal how laying stage and feeding regime interact to shape meat quality and volatile flavor profiles, and to establish differential flavor fingerprints across treatments. The findings will provide scientific support for targeted feeding management and quality standardization of premium Wenchang chicken products.

## 2. Materials and Methods

### 2.1. Experimental Materials and Sample Collection

A 4 × 2 × 2 factorial experimental design was employed, with laying stage (4 levels: 160, 180, 210, and 360 days), feeding regime (2 levels: feed restriction, FR; ad libitum, AL), and muscle type (2 levels: breast muscle; thigh muscle) as fixed factors. Wenchang chicken (*Gallus gallus domesticus*) hens were reared at Longquan Wenchang Chicken Industry Co., Ltd. (Haikou, China). At each of the four laying stages, 6 hens were randomly assigned to either the FR or AL group (3 hens per feeding regime per stage; total *n* = 24 hens). Each hen was treated as an independent biological replicate. From each hen, both breast (pectoralis major) and thigh (iliotibialis) muscles were collected, yielding 48 muscle samples in total (24 hens × 2 muscle cuts). The four age groups were sampled sequentially as the hens reached the target ages, but all samples were stored at −80 °C and subsequently cooked and analyzed in single randomized batches to minimize analytical batch effects. All hens were housed under identical environmental conditions with free access to water.

### 2.2. Coconut Chicken Cooking Procedure

Cooking was performed according to the traditional Hainan coconut chicken recipe. Mature green coconuts (*Cocos nucifera* L.) from the same batch were used to minimize matrix variation. Coconut water was collected and filtered; coconut flesh was peeled and cut into uniform 2 cm × 2 cm pieces.

Frozen muscle samples were thawed overnight at 4 °C, rinsed, and drained. Each sample was cooked at a ratio of 1 g of meat to 5 mL of coconut water, with 5 g of sodium chloride added per liter of coconut broth and no extra seasonings. The coconut broth was brought to a rolling boil, then meat pieces were added and simmered for 15 min. Immediately after cooking, samples were transferred to an ice bath (0–4 °C) for 5 min to terminate thermal reactions. Cooked meat was drained and separated by muscle cut for subsequent analyses.

### 2.3. Meat Quality Determination

Core meat quality traits were determined for both breast and thigh muscles across all treatments, with three technical replicates per sample.

Drip loss: Calculated as the percentage weight loss after 24 h suspension at 4 °C.

Shear force: Measured on cooked meat using a universal texture analyzer (Shanghai TengBa Instrument Technology Co., Ltd., Shanghai, China), expressed in kgf.

pH at 45 min post-mortem: Determined at 45 min post-mortem using a portable pH meter (INESA Scientific Instrument Co., Ltd., Shanghai, China).

Color parameters: Lightness (L*), redness (a*), and yellowness (b*) were recorded with a CR-400 colorimeter (Konica Minolta Sensing, Inc., Tokyo, Japan).

### 2.4. Electronic Nose (E-Nose) Analysis

Overall odor profiling was conducted with an ISENSO Super Nose system following the protocol described in our previous study [14], with sample weight set to 9.00 ± 0.01 g of cooked muscle per 40 mL vial and 30 min headspace equilibration at 25 °C. Principal component analysis (PCA) was performed using the instrument’s built-in software based on the average peak response values of the sensor array.

### 2.5. HS-SPME-GC-MS Analysis

Volatile compounds were extracted and analyzed by headspace solid-phase microextraction coupled with gas chromatography-mass spectrometry. Instrumental parameters, including fiber type, chromatographic column, oven temperature program, and MS conditions, were set as previously reported [14]. Briefly, 3.00 ± 0.01 g of minced cooked muscle was placed in a 20 mL vial, spiked with 1 µL of 0.812 μg/mL 2-methyl-3-heptanone as a system stability control, and equilibrated at 60 °C for 10 min. Extraction was carried out at 60 °C for 30 min.

Compound identification was based on a multi-criteria approach following the Metabolomics Standards Initiative (MSI) confidence levels. For GC-MS analysis, mass spectra were matched against the NIST database with a minimum similarity threshold of 80%. Four key aroma-active aldehydes (hexanal, nonanal, octanal, heptanal) were confirmed by co-injection of authentic reference standards (purity ≥ 95%, Sigma-Aldrich, St. Louis, MO, USA) and are assigned MSI Level 1. All other GC-MS compounds were tentatively identified by MS spectral library matching only and are assigned MSI Level 3 (putatively annotated compound class).

The internal standard (2-methyl-3-heptanone, 0.812 ng per 3.00 g vial) was added to each sample as a system stability and extraction-efficiency control. Volatile composition was analyzed using relative peak area percentages (Area%), and compositional data were further subjected to centered log-ratio (CLR) transformation for multivariate analysis (see Section 2.7). Each sample was analyzed in triplicate (technical replicates), and the mean was used for statistical analysis.

### 2.6. HS-GC-IMS Analysis

Volatile flavor fingerprinting was performed on a FlavourSpec^®^ HS-GC-IMS system (G.A.S. GmbH, Dortmund, Germany). Column and detector parameters were consistent with our established protocol [14]. For each sample, 3.00 ± 0.01 g of cooked muscle was incubated at 60 °C for 15 min before headspace injection. Qualitative identification was achieved by matching retention indices and drift times against the built-in NIST database. Gallery plot fingerprints and PCA clustering were generated using the VOCal 0.4.03 software package.

### 2.7. Statistical Analysis

All data are presented as mean ± standard deviation (SD). The experimental unit for statistical analysis was the individual hen (biological replicate, *n* = 3 per feeding regime per laying stage; total *n* = 48 muscle samples for meat quality traits; *n* = 47 for GC-MS volatile analysis (one 210-day AL thigh replicate was unavailable)). Technical replicates (3 repeated measurements per muscle sample for meat quality traits and GC-MS; 1 injection per sample for GC-IMS) were averaged prior to analysis to avoid pseudo-replication. A three-factor analysis of variance (ANOVA) was performed with laying stage (LS, 4 levels: 160, 180, 210, 360 days), feeding regime (F, 2 levels: FR, AL), and muscle type (M, 2 levels: breast, thigh) as fixed factors, using the model: trait = LS + F + M + LS × F + LS × M + F × M + LS × F × M + *ε*. Type III sum of squares was used. When a significant interaction was detected (*p* < 0.05), simple main effects analysis was conducted, followed by Tukey HSD post hoc tests for pairwise comparisons. Full statistical outputs including effect sizes are provided in Supplementary Table S1 for completeness. For multivariate analysis of the GC-MS volatile profiles, relative peak area data were first subjected to centered log-ratio (CLR) transformation to account for the compositional nature of the data (zero values replaced by half the minimum non-zero value). Principal component analysis (PCA) and partial least squares discriminant analysis (PLS-DA) were then performed on the CLR-transformed data, with separate models fitted for laying stage, feeding regime, and muscle type as response variables. PLS-DA model significance was validated by permutation testing (*n* = 1000 permutations), and variable importance in projection (VIP) scores were calculated to identify the compounds contributing most to group separation. All statistical analyses were performed in R v4.3.2 using the “car”, “emmeans”, “agricolae”, “compositions”, and “mixOmics” packages (all available from CRAN, https://cran.r-project.org). Statistical significance was defined at *p* < 0.05.

## 3. Results

### 3.1. Effects of Laying Age and Feed Restriction on Meat Quality of Wenchang Chicken

Three-way ANOVA revealed that muscle type was the dominant and only significant main effect for meat quality traits, specifically for color parameters: L* (*p* = 0.004), a* (*p* < 0.001), and b* (*p* < 0.001). A significant three-way interaction (age × feeding × muscle) was detected for b* (*p* = 0.040). Shear force, drip loss (0 h and 24 h), and 45 min pH showed no significant main effects or interactions (all *p* > 0.05). Thigh muscle had significantly higher a* and b* values than breast muscle, consistent with its higher myoglobin and intramuscular fat content.

Drip loss (0 h and 24 h) ranged from 4.2% to 5.2% across all treatments and showed no significant differences among laying stages, feeding regimes, or muscle types (all *p* > 0.05, Table 1). Shear force ranged from 2.5 to 7.0 kgf and was not significantly affected by any main effect or interaction (all *p* > 0.05), although numerically higher values were observed at later laying stages. The 45 min post-mortem pH was stable across all treatments (range 6.1–6.9, all *p* > 0.05). For color parameters, thigh muscle consistently showed higher redness (a*) and yellowness (b*) than breast muscle across all age and feeding groups (*p* < 0.001 for both). Detailed values are presented in Table 1 and ANOVA statistics are presented in Table 2.

### 3.2. Overall Volatile Odor Profiles Analyzed by Electronic Nose

**Figure 1:**
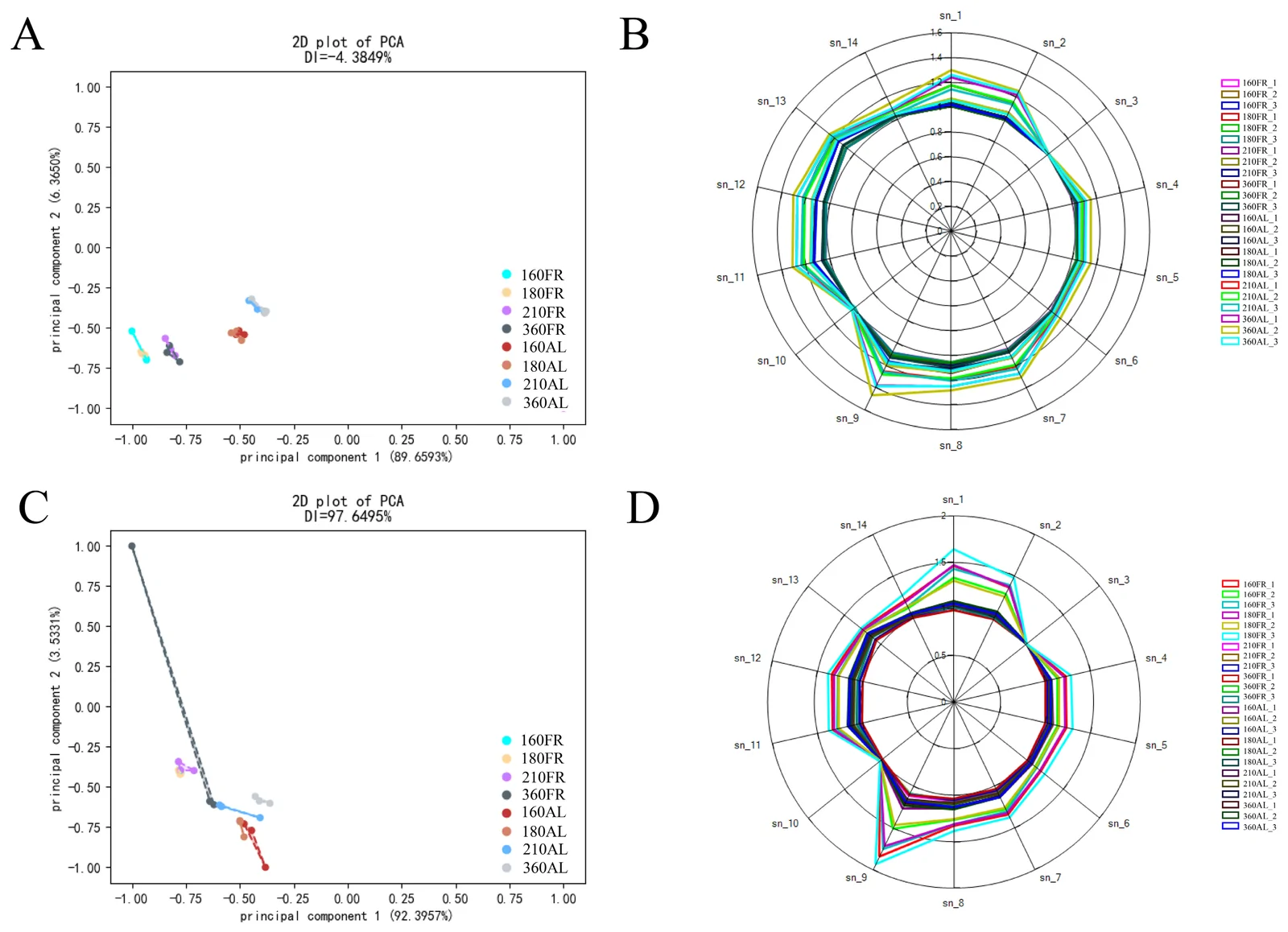
Overall odor profiles of Wenchang chicken muscle analyzed by electronic nose. (**A**) PCA score plot of thigh muscle; (**B**) sensor response radar plot of thigh muscle; (**C**) PCA score plot of breast muscle; (**D**) sensor response radar plot of breast muscle.

For thigh muscle, the PCA score plot (Figure 1A) showed that the first and second principal components explained 89.66% and 6.37% of total variance, respectively, with a cumulative contribution of 96.03%. Samples from different ages showed a gradient distribution along the PC1 axis. The 360-day samples were located at the far left of the PC1 negative axis and were clearly separated from younger age groups. Samples from the FR and AL groups at the same age showed partial overlap but a visible separation trend. The sensor response radar plot (Figure 1B) revealed the highest response values for sn_8 and sn_9 sensors, suggesting that sulfur-containing compounds and aromatic substances may be the main contributors to the overall odor response. Overall response intensities were higher in 360-day samples, and the AL group showed slightly stronger responses than the FR group at the same age.

For breast muscle, the PCA score plot (Figure 1C) showed that PC1 and PC2 accounted for 92.40% and 3.53% of total variance, respectively, with a discrimination index (DI) of 97.65%. The 360-day FR group formed a distinct cluster in the positive PC2 region, far from all other samples. The remaining age groups clustered mainly in the negative PC1 and PC2 regions. The radar plot (Figure 1D) showed consistent response patterns with thigh muscle. The 360-day FR group exhibited stronger responses on multiple sensors (sn_8, sn_9, sn_10), consistent with the PCA distribution.

### 3.3. Volatile Flavor Composition Analyzed by HS-SPME-GC-MS

Volatile compounds in cooked thigh and breast muscles were identified and analyzed by HS-SPME-GC-MS. After removing 91 implausible library artifacts (including fluorinated compounds, flavonoids, pharmaceutical residues, and siloxanes; see Section 2.5), 79 reproducible volatile compounds were detected across all 47 samples, including alkanes, aldehydes, ketones, alcohols, and esters. Three-way ANOVA on individual replicate data revealed that laying stage was a significant main effect for octanal (*p* < 0.001), dodecane (*p* < 0.001), and tetradecane (*p* < 0.001). Critically, the age × feeding interaction was significant for all seven key compounds tested (hexanal: *p* = 0.023; nonanal: *p* = 0.005; octanal: *p* = 0.002; dodecane: *p* < 0.001; undecane: *p* = 0.007; tridecane: *p* = 0.017; tetradecane: *p* < 0.001), formally confirming the stage-dependent reversal of feeding effects. Muscle type was a significant main effect for hexanal (*p* = 0.015) and tridecane (*p* = 0.026). Relative peak area percentages and MSI confidence levels for all identified compounds are presented in Table 3. Full three-way ANOVA results (*F*, *p*, partial *η*^2^*_p_*) for all 79 compounds are provided in Supplementary Table S1.

In thigh muscle, the number of identified compounds ranged from six (210 days) to 28 (180 days) per treatment. At 160 days, eight compounds showed significant FR-AL differences in simple effects tests (*p* < 0.05), with most alkanes and aldehydes (e.g., dodecane, nonanal) showing higher relative abundances in the AL group. At 180 days, seven compounds showed significant intergroup differences, with alkane relative abundances remaining higher in the AL group. At 210 days, only six compounds were detected, with no significant differences between groups. At 360 days, five compounds showed significant differences (*p* < 0.05), and the trend was reversed: most alkanes and aldehydes showed higher relative abundances in the FR group, while trans-2-methylcyclopentanol was significantly higher in the AL group (*p* < 0.05). This reversal is supported by the significant age × feeding interaction in the three-way ANOVA (*p* < 0.001 for dodecane and tetradecane).

In breast muscle, the number of identified compounds ranged from 19 (160 and 360 days) to 36 (210 days), with the highest number at 210 days indicating richer flavor precursors in late-laying breast muscle. At 160 days, two compounds showed significantly higher relative peak area percentages in the AL group (*p* < 0.05). At 180 days, 23 compounds were detected, with no significant intergroup differences after removing implausible entries. At 360 days, trans-2-methylcyclopentanol showed significantly higher relative abundance in the AL group (*p* < 0.05), consistent with the pattern in thigh muscle. The number of significantly differential compounds was lower in breast muscle than in thigh muscle, consistent with the significant muscle-type main effect.

Overall, alkanes, aldehydes, and ketones were the dominant volatile categories in Wenchang chicken muscle. Alkanes, which have high odor thresholds and thus limited direct aroma contribution, are interpreted as indirect indices of lipid-oxidation status. Among aroma-active aldehydes, hexanal showed the highest relative abundance across most treatments, followed by nonanal and octanal. The number of significantly differential compounds was lower in breast muscle than in thigh muscle.

Multivariate analysis on CLR-transformed GC-MS data further supported these findings. PLS-DA revealed significant discrimination among laying stages (R2Y = 0.343, Q2Y = 0.089, permutation *p* < 0.001), feeding regimes (R2Y = 0.915, Q2Y = 0.830, permutation *p* < 0.001), and muscle types (R2Y = 0.621, Q2Y = 0.148, permutation *p* = 0.005). The exceptionally high Q2Y for feeding regime indicates that FR and AL groups have highly distinct volatile profiles, consistent with the significant age × feeding interactions detected by ANOVA. VIP analysis identified cyclopentanol (2-methyl-, trans-), 3-hexanone (2,5-dimethyl-), 4-heptanone (2-methyl-), hexanal, and dodecane as the top contributors to age-related separation (VIP > 1.0). PLS-DA score plots, permutation test results, and VIP score rankings are presented in Supplementary Figure S1.

### 3.4. Identification of Volatile Compounds by HS-GC-IMS

**Figure 2:**
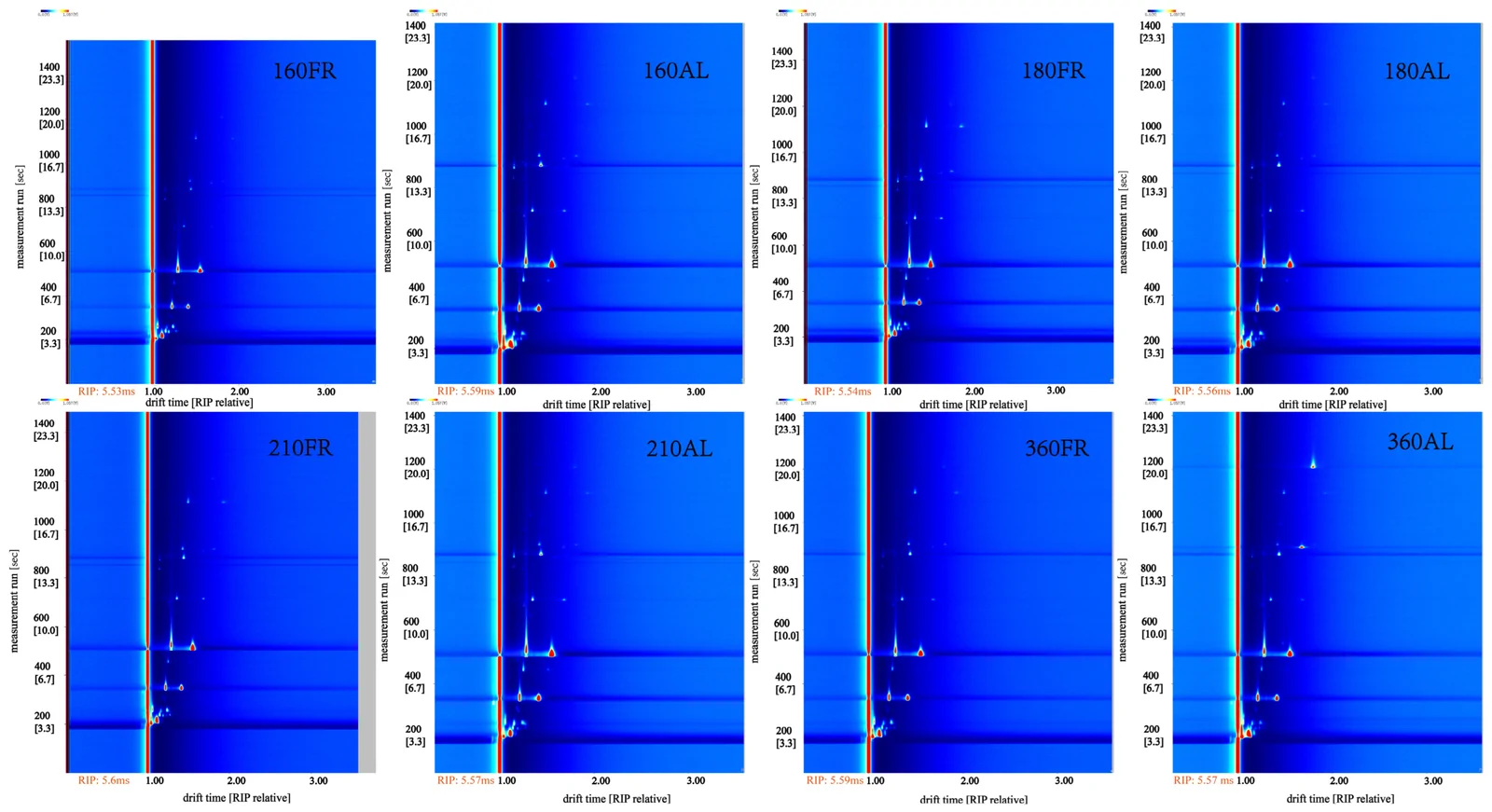
HS-GC-IMS topographic plots of thigh muscle from Wenchang chickens at different laying stages and feeding regimes.

**Figure 3:**
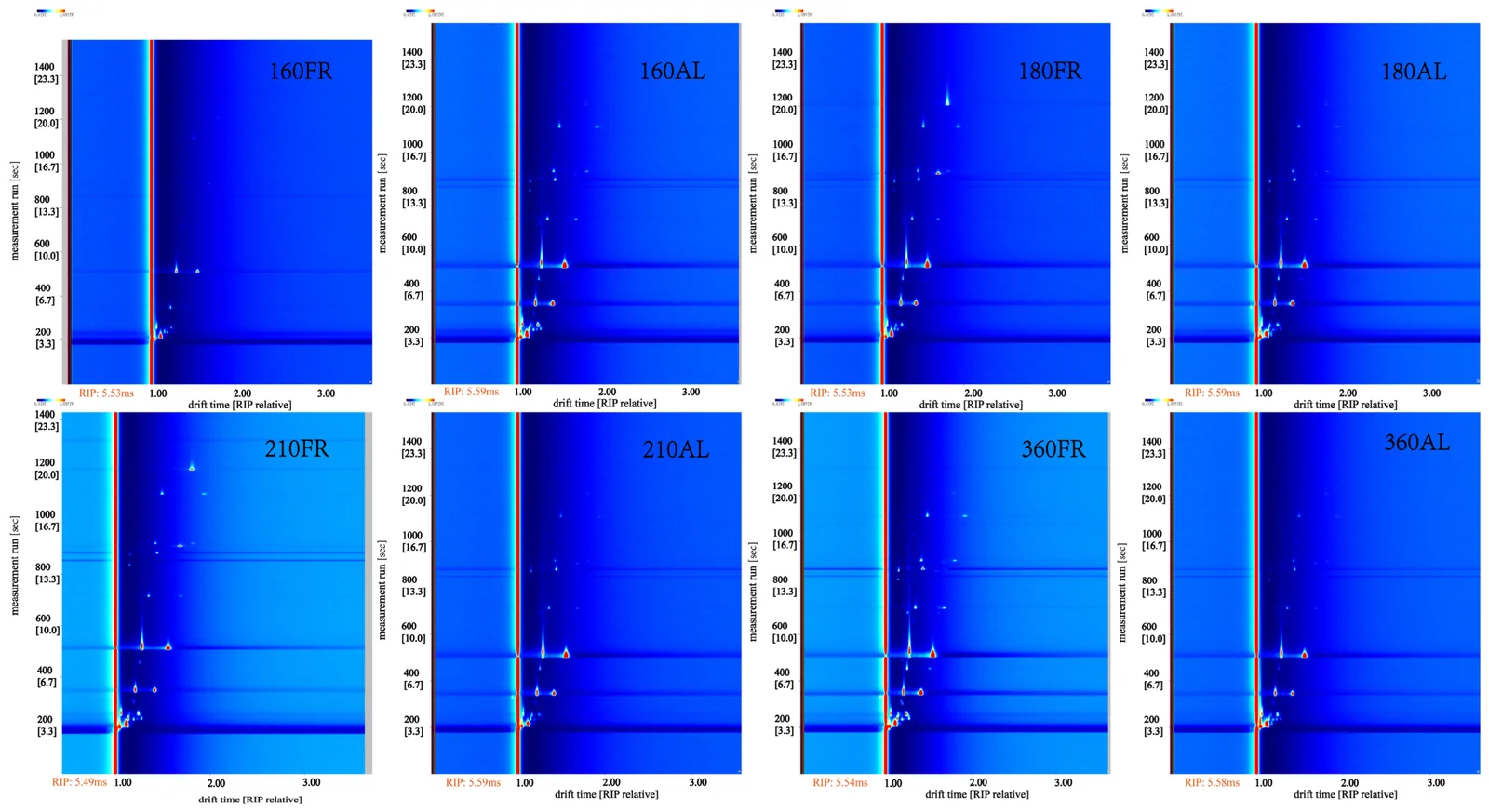
HS-GC-IMS topographic plots of breast muscle from Wenchang chickens at different laying stages and feeding regimes.

HS-GC-IMS was applied to analyze volatile profiles based on two-dimensional separation of gas chromatographic retention time and ion drift time. Typical topographic plots of thigh and breast muscles are presented in Figure 2 and Figure 3, respectively.

All thigh muscle samples shared similar overall topographic features (Figure 2). The vertical red line on the left represents the reactive ion peak (RIP). Signal peaks with varying intensities were distributed to the right of the RIP. Most small-molecule volatiles concentrated in the low retention time region, which contributed predominantly to chicken flavor. With increasing laying age, the number and intensity of signal peaks in the medium-high drift time region increased. The 360-day samples showed more characteristic peaks in the high retention time region. At the same age, the AL group exhibited stronger peak intensities in the medium-low retention time region, especially at 160 and 180 days. Breast muscle showed generally weaker signal intensities and fewer peaks in the high retention time region compared with thigh muscle (Figure 3).

A total of 18 volatile substances were qualitatively identified by matching retention indices and drift times with the NIST database. These compounds covered aldehydes, ketones, alcohols, esters, terpenes, and heterocycles. Some substances existed in both monomer and dimer forms, enriching the flavor dimensionality of chicken meat.

### 3.5. Flavor Fingerprint Analysis

**Figure 4:**
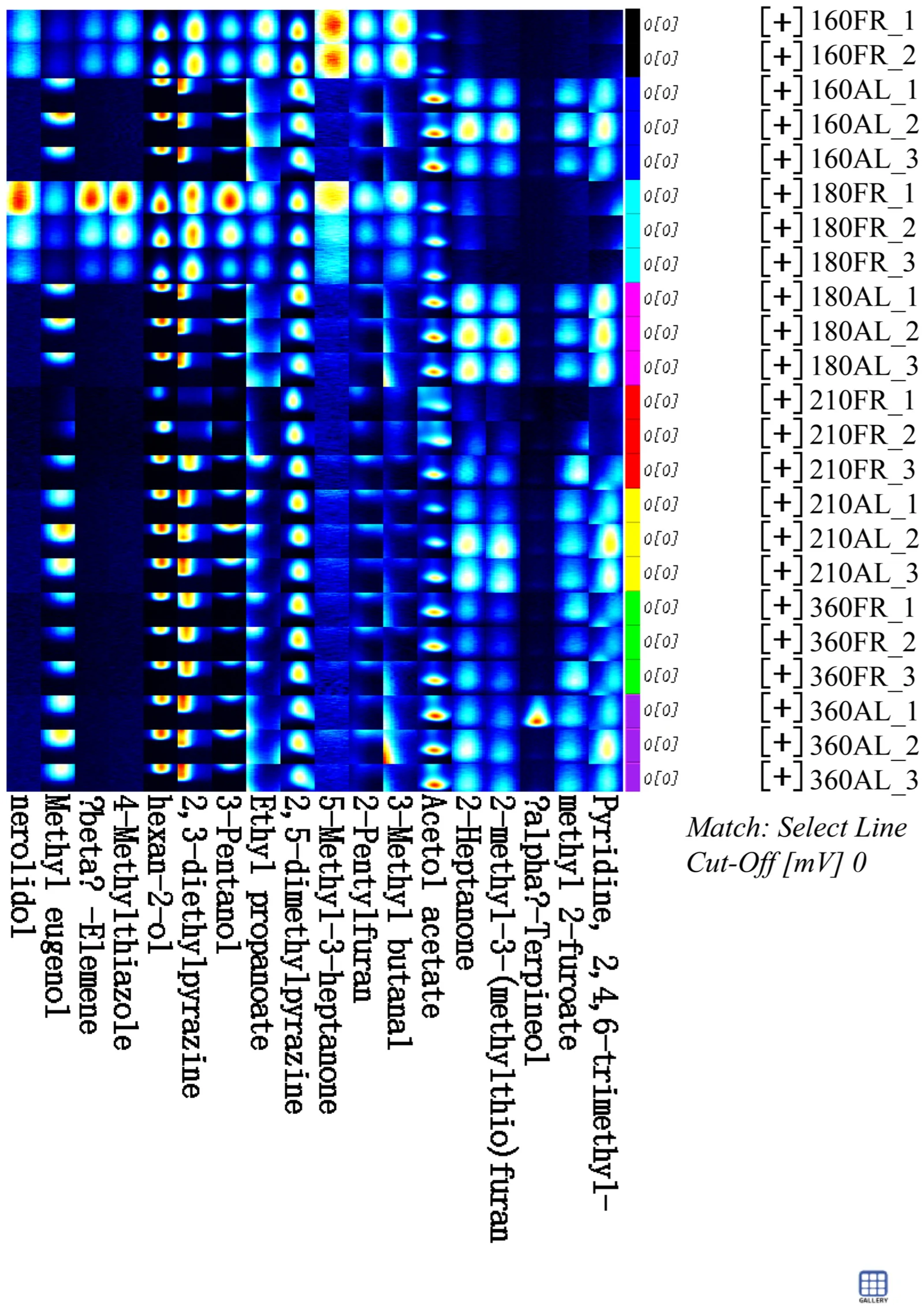
Gallery plot fingerprint of volatile flavor compounds in Wenchang chicken thigh muscle.

**Figure 5:**
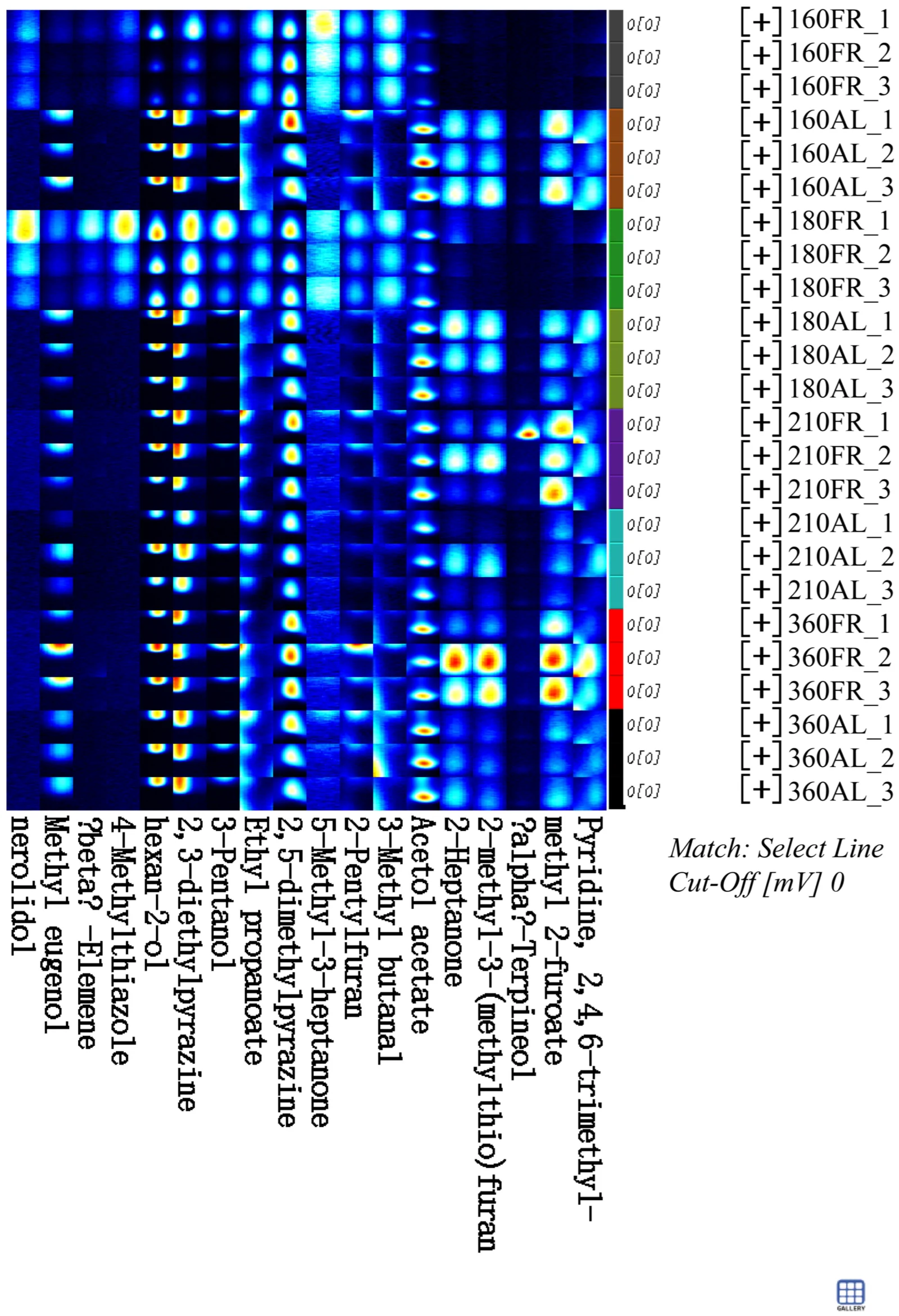
Gallery plot fingerprint of volatile flavor compounds in Wenchang chicken breast muscle.

Volatile flavor fingerprints of all samples were constructed using the Gallery Plot plugin. The results are shown in Figure 4 and Figure 5, respectively.

The fingerprint showed that thigh and breast muscles formed distinct characteristic bands, indicating remarkable differences in their flavor profiles (Figure 4 and Figure 5). Within the same muscle cut, laying age was the primary factor affecting volatile composition. Signals of substances in the high retention index region gradually intensified with increasing age. The effects of feed restriction varied with age: at 160 and 180 days, most aldehydes and esters showed stronger signals in the AL group; at 360 days, this pattern was reversed, with stronger signals in the FR group.

## 4. Discussion

This study systematically reveals the differential regulatory effects of laying age and feed restriction on meat quality traits and volatile flavor profiles of Wenchang chicken breast and thigh muscles. Muscle type is identified as the dominant factor for physical meat quality traits, particularly color parameters [15], while laying stage exerts the strongest influence on volatile flavor compound profiles. The age × feeding interaction is consistently significant across key lipid-oxidation volatiles, indicating that the effects of feed restriction are stage-dependent.

Three-way ANOVA showed that shear force was not significantly affected by laying stage, feeding regime, or muscle type (all *p* > 0.05), although a gradual numerical increase with age was observed. This suggests that within the age range studied (160–360 days), the textural changes associated with advancing physiological maturity do not reach statistical significance, possibly due to the relatively small sample size (*n* = 3 per group) and high within-group variability [16]. The lack of a significant feeding effect on shear force indicates that the moderate feed restriction intensity applied here was insufficient to induce measurable textural changes [17].

Consistent with the ANOVA results, feed restriction did not significantly affect any physical meat quality trait (shear force, drip loss, pH) at any laying stage (all *p* > 0.05) [17]. This finding suggests that the feed restriction intensity applied in this study does not substantially impact the physical properties of Wenchang chicken meat, while its effects on volatile flavor profiles are more pronounced and age-dependent. It is possible that a more severe or longer-duration restriction protocol would yield significant physical quality changes [18,19,20].

At the pre-laying (160 d) and peak-laying (180 d) stages, the relative abundances of lipid oxidation-derived aldehydes and alkanes, such as nonanal, dodecane, and heptadecane, were significantly higher in the AL group than in the FR group. Aldehydes are key contributors to the characteristic flavor of chicken meat, and their formation mainly stems from autoxidation of polyunsaturated fatty acids during thermal processing [2]. Under ad libitum feeding, hens have sufficient energy intake, and the organism tends to store excess energy as intramuscular triglycerides [21,22]. This leads to higher levels of unsaturated fatty acids in muscle, which provide more abundant substrates for lipid oxidation during cooking. Meanwhile, alkanes have high flavor thresholds, but as byproducts or end products of the lipid oxidation chain reaction, changes in their abundance indirectly reflect an increase in the overall oxidation level of muscle [23].

However, this trend was completely reversed at the end-laying stage (360 d): the relative abundances of most differential alkanes and aldehydes were lower in the AL group than in the FR group, while trans-2-methylcyclopentanol was significantly elevated in the AL group. This stage-dependent reversal warrants particular attention. Hens at 360 days are at the end of the laying cycle, with a physiological state fundamentally different from that of younger birds [24]. This finding indicates that the regulatory direction of feed restriction on chicken meat flavor is not constant, but is closely associated with the physiological stage of the birds [20,25].

In this study, thigh and breast muscles formed clearly separate clusters on E-nose PCA score plots, GC-IMS topographic plots, and gallery plot fingerprints. Three-way ANOVA confirmed that muscle type was the dominant main effect for color parameters (L*: *p* = 0.004; a*: *p* < 0.001; b*: *p* < 0.001), with a significant three-way interaction for b* (*p* = 0.040). PLS-DA also showed significant discrimination between breast and thigh muscles (Q2Y = 0.148, permutation *p* = 0.005). Muscle type was the strongest predictor for color traits, while laying stage was the strongest predictor for volatile compounds, followed by the age × feeding interaction. As locomotor muscle supporting continuous weight-bearing movement, thigh muscle contains higher intramuscular fat content and a denser capillary network, which provide abundant substrates and an oxygen-rich environment for lipid oxidation [26]. Thigh muscle also has higher heme iron content, which exerts pro-oxidant activity during thermal processing and effectively catalyzes the autoxidation of unsaturated fatty acids [27]. Consistently, thigh muscle showed stronger volatile signal intensities, a greater number of significantly differential compounds, and a more complex flavor profile compared with breast muscle in the present study.

Under the specific cooking context of coconut chicken, Maillard reactions and Strecker degradation between abundant lipid oxidation products in thigh muscle and sucrose, glucose, and free amino acids in coconut water may be more intense [28,29]. This may represent the chemical basis for the common consumer perception that bone-in thigh meat yields richer and more umami flavor after stewing. Breast muscle presents a lean-meat flavor profile, with its volatile contribution mainly derived from Maillard reactions involving water-soluble precursors [29,30]. This characteristic gives breast muscle independent market value in processed products such as ready-to-eat chicken breast.

Taken together, laying age, feeding regime, and muscle cut exert combined regulatory effects on the coconut chicken-style flavor of Wenchang chicken [3,31]. As the primary factor, laying age exerts effects across all age stages. The impact of feed restriction on flavor is highly dependent on age stage, with no universal promoting or inhibiting effect across the entire production cycle. Differences in fat metabolism and redox microenvironment between thigh and breast muscles form the material basis for their distinct flavor richness [32,33,34].

Despite these contributions, several limitations should be acknowledged when interpreting the results. First, owing to high variability in the response of the internal standard (2-methyl-3-heptanone) across samples, with relative peak areas ranging from 2.5% to 55.7%, reliable absolute semi-quantification of volatile compounds was not achievable. All compositional analyses were therefore performed on relative peak area percentages using compositional data methods (CLR transformation), and conclusions regarding flavor changes refer to relative rather than absolute differences in compound abundance. Furthermore, the study employed three biological replicates per treatment group, which provides moderate statistical power, particularly for detecting three-way interaction effects; some marginally non-significant trends may therefore reflect limited detection sensitivity rather than a true absence of biological effects. Additionally, the findings are specific to Wenchang chickens prepared using the traditional coconut chicken cooking protocol, and generalizability to other poultry breeds or alternative cooking methods—which may modify volatile profiles through differing Maillard reaction and lipid oxidation kinetics—should be made with caution. The intrinsic confounding of laying stage with chronological age in the experimental design also means that observed age-related shifts in flavor cannot be definitively disentangled from general growth and senescence effects. Finally, no descriptive sensory analysis was included, so the perceptual relevance of the identified volatile markers remains inferential.

## 5. Conclusions

This study systematically characterized the meat quality and volatile flavor profiles of Wenchang chicken breast and thigh muscles across different laying stages and feeding regimes, using a multi-technique approach combining E-Nose, HS-SPME-GC-MS, and HS-GC-IMS under traditional coconut chicken cooking conditions. A 4 × 2 × 2 three-way ANOVA was employed to test main effects and interactions.

Three-way ANOVA confirmed laying age as the primary driver of flavor profile differentiation in Wenchang chicken (significant main effect for most volatile categories, *p* < 0.05). Feeding regime was not a significant main effect for physical meat quality traits (all *p* > 0.05), but the age × feeding interaction was significant for several lipid-oxidation-derived volatiles (*p* < 0.001), indicating that feed restriction exerts age-specific regulation on volatile composition. Specifically, ad libitum feeding promotes the formation of lipid-oxidation-derived aldehydes and alkanes at early and middle laying stages, while this effect is completely reversed at the end-laying stage. Muscle type was a significant main effect for color parameters and volatile abundance (*p* < 0.05), with thigh muscle exhibiting stronger volatile signals and more abundant characteristic compounds than breast muscle. Alkanes, which have high odor thresholds, are interpreted as indirect indices of lipid-oxidation status rather than direct aroma contributors; hexanal was the most abundant aroma-active aldehyde across treatments. HS-GC-IMS effectively complements GC-MS in detecting trace polar flavor substances, and the combined use of the two techniques establishes differential flavor fingerprints of Wenchang chicken muscle across varying ages and feeding strategies.

These findings clarify the combined regulation of laying age, feeding regime, and muscle cut on the flavor of coconut chicken-style Wenchang chicken. The results provide a scientific basis for defining the optimal eating quality window and implementing targeted flavor management for specialty Wenchang chicken products.

## Figures and Tables

**Table 1:** Meat quality parameters of Wenchang chicken breast and thigh muscles across four laying stages (160, 180, 210, 360 days) under feed restriction (FR) and ad libitum (AL) feeding. Mean ± SD (*n* = 3 hens per treatment).

Muscle	Trait	160FR	160AL	180FR	180AL	210FR	210AL	360FR	360AL
Thigh	Drip loss (0 h)	4.45 ± 1.04	4.65 ± 0.39	4.56 ± 0.44	4.86 ± 0.23	4.41 ± 0.09	4.56 ± 0.33	4.64 ± 0.08	4.6 ± 0.44
	Drip loss (24 h)	4.33 ± 1	4.53 ± 0.39	4.43 ± 0.4	4.71 ± 0.24	4.31 ± 0.07	4.45 ± 0.32	4.52 ± 0.09	4.49 ± 0.45
	Shear force(kgf)	4.15 ± 0.35	3.78 ± 0.26	3.72 ± 0.8	3.39 ± 0.16	4.44 ± 0.37	4.04 ± 0.34	4.9 ± 0.52	4.41 ± 0.67
	pH (45 min)	6.56 ± 0.09	6.53 ± 0.15	6.63 ± 0.1	6.43 ± 0.12	6.55 ± 0.19	6.32 ± 0.08	6.55 ± 0.11	6.51 ± 0.04
	L*	41.61 ± 0.69	39.99 ± 0.58	38.68 ± 0.8	42.54 ± 3.14	40.75 ± 2.93	39.17 ± 1.08	40.5 ± 0.58	39.61 ± 0.88
	a*	6.95 ± 1.21	8.05 ± 2.68	9.25 ± 2.12	9.39 ± 1.27	10.94 ± 0.85	10.81 ± 2.36	8.58 ± 1.28	10.46 ± 0.61
	b*	8.38 ± 1.22	10.54 ± 0.75	7.75 ± 1.69	11.8 ± 2.16	12.04 ± 2.06	10.54 ± 1.8	9.55 ± 1.32	9.57 ± 0.41
Breast	Drip loss (0 h)	4.86 ± 0.35	4.69 ± 0.13	4.86 ± 0.26	4.82 ± 0.1	4.72 ± 0.12	4.86 ± 0.31	4.69 ± 0.17	4.93 ± 0.17
	Drip loss (24 h)	4.75 ± 0.35	4.57 ± 0.13	4.72 ± 0.28	4.69 ± 0.12	4.6 ± 0.13	4.76 ± 0.29	4.55 ± 0.15	4.84 ± 0.17
	Shear force(kgf)	4.02 ± 0.66	3.75 ± 1.15	4.42 ± 1.78	4.95 ± 1.35	5.06 ± 0.5	5.36 ± 0.85	5.19 ± 2	4.51 ± 0.92
	pH (45 min)	6.51 ± 0.21	6.5 ± 0.34	6.82 ± 0.26	6.5 ± 0.19	6.56 ± 0.23	6.24 ± 0.14	6.54 ± 0.34	6.35 ± 0.38
	L*	45.82 ± 3.47	47.2 ± 5.78	46.26 ± 3.26	47.42 ± 0.74	49.45 ± 2.72	47.1 ± 4.53	44.54 ± 4.17	47.35 ± 2.19
	a*	−0.37 ± 0.52	−1.14 ± 0.45	0.18 ± 0.36	−0.97 ± 0.59	−0.74 ± 0.59	−0.56 ± 0.68	−0.24 ± 0.52	−0.09 ± 0.32
	b*	4.88 ± 0.43	5.66 ± 0.9	5.57 ± 0.25	5.21 ± 0.48	6.39 ± 0.55	6.44 ± 1.81	5.84 ± 1.35	6.47 ± 1.1

**Table 2:** Meat quality parameters of Wenchang chicken breast and thigh muscles across four laying stages (160, 180, 210, 360 days) under feed restriction (FR) and ad libitum (AL) feeding. Three-way ANOVA results (F-value and *p*-value; * *p* < 0.05).

Trait	Age	Feeding	Muscle	Age × Feed	Age × Muscle	Feed × Muscle	3-Way
Drip loss (0 h)	0.24 (*p* = 0.870)	0.34 (*p* = 0.562)	0.01 (*p* = 0.903)	0.39 (*p* = 0.763)	0.40 (*p* = 0.757)	0.79 (*p* = 0.382)	0.53 (*p* = 0.663)
Drip loss (24 h)	0.30 (*p* = 0.823)	0.38 (*p* = 0.542)	0.02 (*p* = 0.881)	0.49 (*p* = 0.688)	0.40 (*p* = 0.756)	0.85 (*p* = 0.362)	0.59 (*p* = 0.624)
Shear force (kgf)	1.59 (*p* = 0.211)	0.12 (*p* = 0.726)	0.00 (*p* = 0.973)	0.51 (*p* = 0.679)	1.11 (*p* = 0.359)	0.01 (*p* = 0.926)	0.21 (*p* = 0.892)
PH (45 min)	1.10 (*p* = 0.365)	0.00 (*p* = 0.964)	0.03 (*p* = 0.872)	0.76 (*p* = 0.527)	0.32 (*p* = 0.811)	0.01 (*p* = 0.942)	0.10 (*p* = 0.962)
L*	0.01 (*p* = 0.999)	0.35 (*p* = 0.556)	9.67 (*p* = 0.004 *)	0.89 (*p* = 0.455)	0.37 (*p* = 0.776)	0.83 (*p* = 0.368)	0.87 (*p* = 0.468)
a*	0.41 (*p* = 0.744)	0.56 (*p* = 0.459)	80.49 (*p* = <0.001 *)	0.42 (*p* = 0.737)	0.77 (*p* = 0.522)	1.68 (*p* = 0.205)	0.48 (*p* = 0.699)
b*	0.68 (*p* = 0.568)	0.54 (*p* = 0.469)	21.32 (*p* = <0.001 *)	0.25 (*p* = 0.860)	1.95 (*p* = 0.142)	0.86 (*p* = 0.361)	3.11 (*p* = 0.040 *)

**Table 3:** Volatile compounds identified by HS-SPME-GC-MS in thigh and breast muscle of Wenchang chicken at different laying stages. Values are mean ± SD of relative peak area (%). MSI confidence levels: 1 = confirmed by authentic standard (hexanal, nonanal, octanal, heptanal); 3 = tentatively identified by MS library match only (≥80% similarity). The *p*-values shown are from independent-samples *t*-tests comparing FR vs. AL within each laying stage. Full three-way ANOVA results are in Supplementary Table S1.

Age	Cas	Compound	Formula	FR Chicken (Area %)	AL Chicken (Area %)	*p*_Value	Fold Change (FR/AL)	MSI Level
Thigh 160 days	1002-43-3	Undecane, 3-methyl-	C_12_H_26_	0.11 ± 0.05	0.64 ± 0.22	0.0478	−2.5406	3
	1120-21-4	Undecane	C_11_H_24_	2.27 ± 0.37	2.93 ± 0.22	0.0686	−0.372	3
	112-40-3	Dodecane	C_12_H_26_	4.52 ± 1.19	11.72 ± 0.56	0.003	−1.3742	3
	124-19-6	Nonanal	C_9_H_18_O	1.46 ± 0.28	2.37 ± 0.24	0.0128	−0.7002	1
	13019-20-0	3-Heptanone, 2-methyl-	C_8_H_16_O	20.27 ± 11.31	11.42 ± 3.63	0.307	0.828	3
	17301-23-4	Undecane, 2,6-dimethyl-	C_13_H_28_	0.38 ± 0.08	0.67 ± 0.22	0.1386	−0.838	3
	1888-57-9	3-Hexanone, 2,5-dimethyl-	C_8_H_16_O	8.98 ± 7.51	18.9 ± 9.95	0.2452	−1.0736	3
	26741-18-4	9-methylheptadecane	C_18_H_38_	0.06 ± 0.01	0.26 ± 0.06	0.109	−2.1155	3
	626-33-5	4-Heptanone, 2-methyl-	C_8_H_16_O	14.13 ± 1.9	3.64 ± 6.28	0.0909	1.9568	3
	629-50-5	Tridecane	C_13_H_28_	2.22 ± 0.97	6.12 ± 4.26	0.2506	−1.4644	3
	629-59-4	Tetradecane	C_14_H_30_	0.68 ± 0.16	2.48 ± 0.57	0.0246	−1.8719	3
	6418-41-3	Tridecane, 3-methyl-	C_14_H_30_	0.2 ± 0.07	0.71 ± 0.12	0.0065	−1.8346	3
	66-25-1	Hexanal	C_6_H_12_O	70.05 ± 41.33	86.83 ± 23.07	0.5809	−0.3098	1
	13151-34-3	Decane, 3-methyl-	C_11_H_24_	0.03 ± 0.03	0.9 ± 0.32	0.042	−4.9015	3
	17312-77-5	Undecane, 2,3-dimethyl-	C_13_H_28_	0.06 ± 0.01	0.22 ± 0.18	0.4117	−1.9069	3
	3891-98-3	Dodecane, 2,6,10-trimethyl-	C_15_H_32_	0.08 ± 0.04	0.72 ± 0.45	0.1286	−3.1766	3
	54833-48-6	Heptadecane, 2,6,10,15-tetramethyl-	C_21_H_44_	0.1 ± 0.01	0.38 ± 0.04	0.0468	−2.0189	3
	62108-27-4	Decane, 2,4,6-trimethyl-	C_13_H_28_	0.12 ± 0.03	0.18 ± 0.04	0.2559	−0.585	3
Thigh 180 days	1120-21-4	Undecane	C_11_H_24_	2.92 ± 0.62	2.06 ± 0.94	0.2655	0.5033	3
	112-40-3	Dodecane	C_12_H_26_	4.1 ± 1.02	12.37 ± 3.07	0.0325	−1.5931	3
	124-19-6	Nonanal	C_9_H_18_O	2.38 ± 1.21	1.27 ± 1.1	0.3048	0.9082	1
	13019-20-0	3-Heptanone, 2-methyl-	C_8_H_16_O	18.43 ± 5.25	17.84 ± 8.44	0.9235	0.0472	3
	17301-23-4	Undecane, 2,6-dimethyl-	C_13_H_28_	0.47 ± 0.1	1.29 ± 0.35	0.0477	−1.4529	3
	17312-77-5	Undecane, 2,3-dimethyl-	C_13_H_28_	0.14 ± 0.04	0.34 ± 0.12	0.0864	−1.2801	3
	1888-57-9	3-Hexanone, 2,5-dimethyl-	C_8_H_16_O	7.7 ± 6.54	8.57 ± 7.32	0.8855	−0.1545	3
	25144-04-1	Cyclopentanol, 2-methyl-, trans-	C_6_H_12_O	0.08 ± 0.05	0.13 ± 0.08	0.4063	−0.737	3
	26741-18-4	9-methylheptadecane	C_18_H_38_	0.14 ± 0.06	0.3 ± 0.08	0.1622	−1.0753	3
	3891-98-3	Dodecane, 2,6,10-trimethyl-	C_15_H_32_	0.19 ± 0.07	0.45 ± 0.16	0.0895	−1.2332	3
	62108-23-0	Decane, 2,5,6-trimethyl-	C_13_H_28_	0.04 ± 0.06	0.32 ± 0.22	0.1463	−3.015	3
	62108-25-2	Decane, 2,6,7-trimethyl-	C_13_H_28_	0.05 ± 0.04	0.78 ± 0.47	0.1149	−3.9573	3
	626-33-5	4-Heptanone, 2-methyl-	C_8_H_16_O	7.28 ± 6.3	13.06 ± 0.48	0.2524	−0.8435	3
	629-50-5	Tridecane	C_13_H_28_	1.84 ± 1.02	2.98 ± 2.34	0.5023	−0.6914	3
	629-59-4	Tetradecane	C_14_H_30_	0.9 ± 0.06	2.17 ± 0.51	0.048	−1.2719	3
	6418-41-3	Tridecane, 3-methyl-	C_14_H_30_	0.22 ± 0.12	0.55 ± 0.04	0.0348	−1.3132	3
	66-25-1	Hexanal	C_6_H_12_O	82.46 ± 39.11	111.25 ± 9.41	0.3296	−0.432	1
	74645-98-0	Dodecane, 2,7,10-trimethyl-	C_15_H_32_	0.16 ± 0.01	0.2 ± 0.04	0.4371	−0.241	3
	1002-43-3	Undecane, 3-methyl-	C_12_H_26_	0.23 ± 0.07	0.5 ± 0.39	0.3537	−1.1106	3
	10374-74-0	7-Tetradecene	C_14_H_28_	0.18 ± 0.03	0.06 ± 0.01	0.0854	1.7105	3
	2425-77-6	1-Decanol, 2-hexyl-	C_16_H_34_O	0.17 ± 0.01	0.52 ± 0.39	0.2622	−1.613	3
	124-13-0	Octanal	C_8_H_16_O	0.42 ± 0.21	0.53 ± 0.4	0.7328	−0.3094	1
	2847-72-5	Decane, 4-methyl-	C_11_H_24_	0.01 ± 0.01	0.46 ± 0.16	0.0411	−4.9281	3
	62108-26-3	Decane, 2,6,8-trimethyl-	C_13_H_28_	0.17 ± 0.04	0.59 ± 0.68	0.5421	−1.7952	3
	629-78-7	Heptadecane	C_17_H_36_	0.03 ± 0.03	0.43 ± 0.04	0.0013	−3.8524	3
	629-92-5	Nonadecane	C_19_H_40_	0.05 ± 0.06	0.46 ± 0.51	0.4571	−3.2016	3
Thigh 210 days	112-40-3	Dodecane	C_12_H_26_	9.52 ± 4.43	0.11 ± 0.06	0.0667	6.4354	3
	13019-20-0	3-Heptanone, 2-methyl-	C_8_H_16_O	35.14 ± 20.72	3.3 ± 1.18	0.1163	3.4103	3
	1888-57-9	3-Hexanone, 2,5-dimethyl-	C_8_H_16_O	5.12 ± 7.08	1.09 ± 1.33	0.4297	2.2328	3
	62108-23-0	Decane, 2,5,6-trimethyl-	C_13_H_28_	0.18 ± 0.03	0.12 ± 0.04	0.1735	0.6464	3
	62108-25-2	Decane, 2,6,7-trimethyl-	C_13_H_28_	0.25 ± 0.22	0.22 ± 0.16	0.8664	0.1982	3
	66-25-1	Hexanal	C_6_H_12_O	12.53 ± 8.78	55.73 ± 40.76	0.3687	−2.1531	1
Thigh 360 days	124-19-6	Nonanal	C_9_H_18_O	2.71 ± 0.78	0.76 ± 0.17	0.0435	1.8342	1
	14676-29-0	Heptane, 3-ethyl-2-methyl-	C_10_H_22_	0.3 ± 0.02	0.26 ± 0.13	0.7048	0.2303	3
	17301-23-4	Undecane, 2,6-dimethyl-	C_13_H_28_	0.74 ± 0.06	0.24 ± 0.09	0.0029	1.5981	3
	17312-63-9	Nonane, 5-butyl-	C_13_H_28_	0.24 ± 0.06	0.35 ± 0.04	0.103	−0.5443	3
	18344-37-1	Heptadecane, 2,6,10,14-tetramethyl-	C_21_H_44_	0.26 ± 0.09	0.06 ± 0.04	0.0439	2.241	3
	1888-57-9	3-Hexanone, 2,5-dimethyl-	C_8_H_16_O	9.05 ± 7.75	14.65 ± 12.55	0.5532	−0.6952	3
	25144-04-1	Cyclopentanol, 2-methyl-, trans-	C_6_H_12_O	0.04 ± 0.04	1.58 ± 0.16	0.0022	−5.1913	3
	54105-67-8	Heptadecane, 2,6-dimethyl-	C_19_H_40_	0.21 ± 0.2	0.28 ± 0.21	0.7561	−0.389	3
	17257-82-8	2-Butanone, 3,4-epoxy-3-ethyl-	C_6_H_10_O_2_	0.56 ± 0.02	0.32 ± 0.05	0.0545	0.8429	3
	3891-98-3	Dodecane, 2,6,10-trimethyl-	C_15_H_32_	0.37 ± 0	0.05 ± 0.03	0.0021	2.7944	3
Breast 160 days	1002-43-3	Undecane, 3-methyl-	C_12_H_26_	0.34 ± 0.24	1 ± 0.22	0.0266	−1.5516	3
	1120-21-4	Undecane	C_11_H_24_	4.18 ± 2.16	3.31 ± 1.57	0.6066	0.3352	3
	112-40-3	Dodecane	C_12_H_26_	5.12 ± 1.75	16.43 ± 1.46	0.0012	−1.6824	3
	17301-23-4	Undecane, 2,6-dimethyl-	C_13_H_28_	0.77 ± 0.17	1.34 ± 0.61	0.2403	−0.7967	3
	124-19-6	Nonanal	C_9_H_18_O	1.43 ± 0.12	2.04 ± 0.58	0.2083	−0.5126	1
	13019-20-0	3-Heptanone, 2-methyl-	C_8_H_16_O	22.08 ± 15.39	12.23 ± 3.74	0.3839	0.8521	3
	1888-57-9	3-Hexanone, 2,5-dimethyl-	C_8_H_16_O	10.85 ± 10.79	11.99 ± 3.59	0.8758	−0.1437	3
	2425-77-6	1-Decanol, 2-hexyl-	C_16_H_34_O	0.43 ± 0.19	4.21 ± 6.41	0.4145	−3.3071	3
	3891-98-3	Dodecane, 2,6,10-trimethyl-	C_15_H_32_	0.71 ± 0.13	2.15 ± 2.58	0.4333	−1.6075	3
	62108-26-3	Decane, 2,6,8-trimethyl-	C_13_H_28_	0.27 ± 0.08	0.62 ± 0.33	0.3611	−1.2109	3
	629-50-5	Tridecane	C_13_H_28_	4.29 ± 2.81	2.68 ± 1.34	0.4386	0.6805	3
	629-59-4	Tetradecane	C_14_H_30_	3.36 ± 1.74	1.66 ± 0.42	0.2281	1.0173	3
	629-62-9	Pentadecane	C_15_H_32_	0.12 ± 0.12	0.24 ± 0.05	0.245	−0.9696	3
	629-92-5	Nonadecane	C_19_H_40_	0.24 ± 0.05	2.06 ± 2.89	0.3897	−3.0816	3
	6418-41-3	Tridecane, 3-methyl-	C_14_H_30_	0.48 ± 0.07	0.85 ± 0.59	0.3865	−0.8301	3
	66-25-1	Hexanal	C_6_H_12_O	29.06 ± 6.68	31.77 ± 13.04	0.7695	−0.1288	1
	17312-77-5	Undecane, 2,3-dimethyl-	C_13_H_28_	0.16 ± 0.09	0.67 ± 0.26	0.0638	−2.1047	3
	544-76-3	Hexadecane	C_16_H_34_	0.64 ± 0.62	0.27 ± 0.14	0.5478	1.2386	3
	626-33-5	4-Heptanone, 2-methyl-	C_8_H_16_O	19.6 ± 2.88	11.95 ± 3.58	0.0875	0.7138	3
	629-78-7	Heptadecane	C_17_H_36_	0.13 ± 0.11	1.19 ± 1.38	0.314	−3.1984	3
Breast 180 days	1002-43-3	Undecane, 3-methyl-	C_12_H_26_	0.31 ± 0.37	0.58 ± 0.35	0.4111	−0.8966	3
	1120-21-4	Undecane	C_11_H_24_	2.61 ± 1.35	1.85 ± 0.69	0.4534	0.4921	3
	112-40-3	Dodecane	C_12_H_26_	5.1 ± 5.9	11.78 ± 1.96	0.1805	−1.2078	3
	124-19-6	Nonanal	C_9_H_18_O	1.63 ± 0.85	2.3 ± 0.32	0.3081	−0.4947	1
	13019-20-0	3-Heptanone, 2-methyl-	C_8_H_16_O	16.53 ± 14.57	21.81 ± 11.82	0.6526	−0.4	3
	17301-23-4	Undecane, 2,6-dimethyl-	C_13_H_28_	0.64 ± 0.06	0.52 ± 0.44	0.6944	0.2903	3
	18344-37-1	Heptadecane, 2,6,10,14-tetramethyl-	C_21_H_44_	0.42 ± 0.08	1.57 ± 0.51	0.0564	−1.8852	3
	1888-57-9	3-Hexanone, 2,5-dimethyl-	C_8_H_16_O	2.46 ± 2.19	3.91 ± 6.74	0.751	−0.6705	3
	25144-04-1	Cyclopentanol, 2-methyl-, trans-	C_6_H_12_O	1.43 ± 1.23	0.02 ± 0.03	0.1844	5.9408	3
	26741-18-4	9-methylheptadecane	C_18_H_38_	0.32 ± 0.35	0.14 ± 0.08	0.578	1.2675	3
	31295-56-4	Dodecane, 2,6,11-trimethyl-	C_15_H_32_	0.29 ± 0.16	0.55 ± 0.35	0.4782	−0.9234	3
	3891-98-3	Dodecane, 2,6,10-trimethyl-	C_15_H_32_	0.96 ± 0.74	3.29 ± 1.99	0.1728	−1.777	3
	544-76-3	Hexadecane	C_16_H_34_	0.68 ± 0	3.68 ± 2.58	0.1821	−2.4348	3
	54833-48-6	Heptadecane, 2,6,10,15-tetramethyl-	C_21_H_44_	0.5 ± 0.37	0.42 ± 0.32	0.8399	0.2543	3
	62108-23-0	Decane, 2,5,6-trimethyl-	C_13_H_28_	0.47 ± 0.08	0.25 ± 0.16	0.2564	0.9107	3
	62108-25-2	Decane, 2,6,7-trimethyl-	C_13_H_28_	0.24 ± 0.3	0.96 ± 0.86	0.2894	−1.9652	3
	629-50-5	Tridecane	C_13_H_28_	2.54 ± 0.11	1.99 ± 0.96	0.4265	0.3516	3
	629-59-4	Tetradecane	C_14_H_30_	1.98 ± 0.5	4.59 ± 1.45	0.0755	−1.2156	3
	629-78-7	Heptadecane	C_17_H_36_	0.98 ± 0.71	2.88 ± 1.38	0.1391	−1.5495	3
	629-92-5	Nonadecane	C_19_H_40_	1.81 ± 1.43	3.88 ± 1.95	0.2695	−1.1001	3
	6418-41-3	Tridecane, 3-methyl-	C_14_H_30_	0.38 ± 0.3	0.84 ± 0.26	0.2265	−1.1255	3
	66-25-1	Hexanal	C_6_H_12_O	30.82 ± 21.79	86.84 ± 19.41	0.096	−1.4945	1
Breast 210 days	1002-43-3	Undecane, 3-methyl-	C_12_H_26_	1.7 ± 1.04	0.55 ± 0.42	0.1871	1.628	3
	1120-21-4	Undecane	C_11_H_24_	8.08 ± 4.92	2.28 ± 2.88	0.1951	1.8291	3
	112-40-3	Dodecane	C_12_H_26_	17.43 ± 7.59	13.06 ± 4.48	0.4495	0.416	3
	13019-16-4	2-Octenal, 2-butyl-	C_12_H_22_O	0.32 ± 0.28	1.05 ± 0.35	0.0869	−1.737	3
	13019-20-0	3-Heptanone, 2-methyl-	C_8_H_16_O	15.63 ± 9.77	32.42 ± 8.32	0.0877	−1.0527	3
	13151-34-3	Decane, 3-methyl-	C_11_H_24_	0.17 ± 0.03	2.35 ± 1.34	0.1063	−3.7891	3
	17301-23-4	Undecane, 2,6-dimethyl-	C_13_H_28_	1.21 ± 0.95	1.83 ± 0.91	0.5341	−0.5995	3
	17302-36-2	5-Ethyldecane	C_12_H_26_	0.28 ± 0.12	0.6 ± 0.2	0.22	−1.0825	3
	1888-57-9	3-Hexanone, 2,5-dimethyl-	C_8_H_16_O	0.91 ± 1.38	3.99 ± 6.63	0.5077	−2.1284	3
	2425-77-6	1-Decanol, 2-hexyl-	C_16_H_34_O	2.84 ± 3.04	2.18 ± 1.26	0.7554	0.3794	3
	25117-24-2	Tetradecane, 4-methyl-	C_15_H_32_	0.52 ± 0.2	0.46 ± 0.22	0.7744	0.1572	3
	3891-98-3	Dodecane, 2,6,10-trimethyl-	C_15_H_32_	1.96 ± 1.73	1.36 ± 0.63	0.618	0.5272	3
	3891-99-4	2,6,10-Trimethyltridecane	C_16_H_34_	0.88 ± 0.88	0.21 ± 0.06	0.3154	2.1019	3
	54105-67-8	Heptadecane, 2,6-dimethyl-	C_19_H_40_	0.24 ± 0.02	1.33 ± 0.92	0.1743	−2.4905	3
	544-76-3	Hexadecane	C_16_H_34_	0.83 ± 1.39	1.29 ± 0.55	0.6349	−0.6362	3
	54833-48-6	Heptadecane, 2,6,10,15-tetramethyl-	C_21_H_44_	0.32 ± 0.25	0.92 ± 0.73	0.2947	−1.5012	3
	55162-61-3	Tetracontane, 3,5,24-trimethyl-	C_43_H_88_	0.84 ± 0.23	0.48 ± 0.09	0.1017	0.7981	3
	56797-40-1	7-Hexadecenal, (Z)-	C_16_H_30_O	0.28 ± 0.22	1.06 ± 0.4	0.0678	−1.9466	3
	62108-23-0	Decane, 2,5,6-trimethyl-	C_13_H_28_	0.27 ± 0.35	0.86 ± 1.22	0.615	−1.6797	3
	62108-25-2	Decane, 2,6,7-trimethyl-	C_13_H_28_	0.33 ± 0.17	1.34 ± 1.31	0.3128	−2.0217	3
	62108-26-3	Decane, 2,6,8-trimethyl-	C_13_H_28_	0.27 ± 0.01	0.93 ± 0.97	0.3594	−1.7894	3
	62108-27-4	Decane, 2,4,6-trimethyl-	C_13_H_28_	0.23 ± 0.07	0.9 ± 0.81	0.2846	−1.9736	3
	626-33-5	4-Heptanone, 2-methyl-	C_8_H_16_O	10.29 ± 2.25	12.08 ± 10.65	0.8015	−0.2318	3
	629-50-5	Tridecane	C_13_H_28_	2.78 ± 1.38	1.65 ± 0.41	0.2886	0.7526	3
	629-59-4	Tetradecane	C_14_H_30_	2.66 ± 0.21	3.35 ± 1.44	0.4914	−0.336	3
	629-92-5	Nonadecane	C_19_H_40_	1.34 ± 1.72	1.17 ± 0.27	0.8833	0.1921	3
	6418-41-3	Tridecane, 3-methyl-	C_14_H_30_	0.61 ± 0.32	0.65 ± 0.2	0.8749	−0.0842	3
	66-25-1	Hexanal	C_6_H_12_O	51.05 ± 30.31	36.49 ± 22.71	0.5445	0.4845	1
	124-18-5	Decane	C_10_H_22_	2.75 ± 1.46	4.23 ± 1.57	0.4319	−0.6212	3
	18344-37-1	Heptadecane, 2,6,10,14-tetramethyl-	C_21_H_44_	0.16 ± 0.21	2.44 ± 1.42	0.1041	−3.9785	3
	25117-31-1	Tridecane, 5-methyl-	C_14_H_30_	0.09 ± 0.06	0.06 ± 0.01	0.7338	0.387	3
	26741-18-4	9-methylheptadecane	C_18_H_38_	1.02 ± 0.61	0.47 ± 0.06	0.4211	1.1178	3
	629-62-9	Pentadecane	C_15_H_32_	0.5 ± 0.4	0.7 ± 0.29	0.6243	−0.4711	3
	629-78-7	Heptadecane	C_17_H_36_	3.09 ± 3.71	1.19 ± 0.84	0.5991	1.379	3
Breast 360 days	13019-20-0	3-Heptanone, 2-methyl-	C_8_H_16_O	23.42 ± 11.22	6.49 ± 1.98	0.1163	1.852	3
	13151-34-3	Decane, 3-methyl-	C_11_H_24_	0.32 ± 0.46	0.3 ± 0.23	0.946	0.1316	3
	17301-23-4	Undecane, 2,6-dimethyl-	C_13_H_28_	0.94 ± 0.47	0.4 ± 0.2	0.1738	1.2327	3
	17302-36-2	5-Ethyldecane	C_12_H_26_	0.27 ± 0.04	0.06 ± 0.09	0.1521	2.0544	3
	18344-37-1	Heptadecane, 2,6,10,14-tetramethyl-	C_21_H_44_	0.38 ± 0.04	0.62 ± 0.3	0.4661	−0.7073	3
	1888-57-9	3-Hexanone, 2,5-dimethyl-	C_8_H_16_O	9.91 ± 8.53	10.85 ± 5.91	0.883	−0.1317	3
	3891-98-3	Dodecane, 2,6,10-trimethyl-	C_15_H_32_	0.45 ± 0.05	0.47 ± 0.57	0.9682	−0.0627	3
	62108-23-0	Decane, 2,5,6-trimethyl-	C_13_H_28_	0.03 ± 0.01	0.29 ± 0.1	0.1628	−3.5361	3
	62108-25-2	Decane, 2,6,7-trimethyl-	C_13_H_28_	0.26 ± 0.11	0.51 ± 0.04	0.0495	−0.9536	3
	626-33-5	4-Heptanone, 2-methyl-	C_8_H_16_O	5.2 ± 7.34	0.03 ± 0.04	0.5009	7.7004	3
	629-59-4	Tetradecane	C_14_H_30_	1.98 ± 0.9	0.42 ± 0.42	0.0811	2.2543	3
	629-78-7	Heptadecane	C_17_H_36_	0.3 ± 0.3	0.19 ± 0.1	0.5907	0.6749	3
	66-25-1	Hexanal	C_6_H_12_O	64.58 ± 41.35	61.06 ± 9.33	0.8984	0.081	1
	25144-04-1	Cyclopentanol, 2-methyl-, trans-	C_6_H_12_O	0.04 ± 0.06	1.01 ± 0.1	0.001	−4.493	3
	62108-27-4	Decane, 2,4,6-trimethyl-	C_13_H_28_	0.17 ± 0.04	0.28 ± 0.19	0.5629	−0.7036	3

## Data Availability

The original contributions presented in this study are included in the article/Supplementary Materials. Further inquiries can be directed to the corresponding author.

## Supplementary Materials

The following supporting information can be downloaded at: https://www.mdpi.com/article/10.3390/foods15183326/s1, Figure S1: Multivariate analysis of GC-MS volatile profiles. (**A**) PLS-DA score plots colored by laying stage, feeding regime, and muscle type (CLR-transformed data, 47 samples × 79 compounds). (**B**) Permutation test results (*n* = 1000) for each PLS-DA model, showing observed R2Y/Q2Y (dashed lines) against permuted distributions. (**C**) VIP score bar plot for the top 20 compounds contributing to laying-stage discrimination; Table S1: Three-way ANOVA results (F-value, *p*-value, partial η^2^*p*) for all 79 reproducible volatile compounds identified by HS-SPME-GC-MS, with relative peak area (%) as the dependent variable. Results are shown for the main effects (age, feeding, muscle) and all interaction effects.
