# Supplementary material for: Laying Stage-Modulated Effects of Feed Restriction on Volatile Compounds of Wenchang Chicken Muscle Assessed by E-Nose, GC-MS and GC-IMS
Source: Foods. 2026 Sep 19;15(18):3326. doi: 10.3390/foods15183326 (PMC13606559; doi:10.3390/foods15183326)
Supplement: Supplementary file 1 [file foods-15-03326-s001.zip › foods-4509973-Supplementary Figure S1.pdf]

foods

MDPI

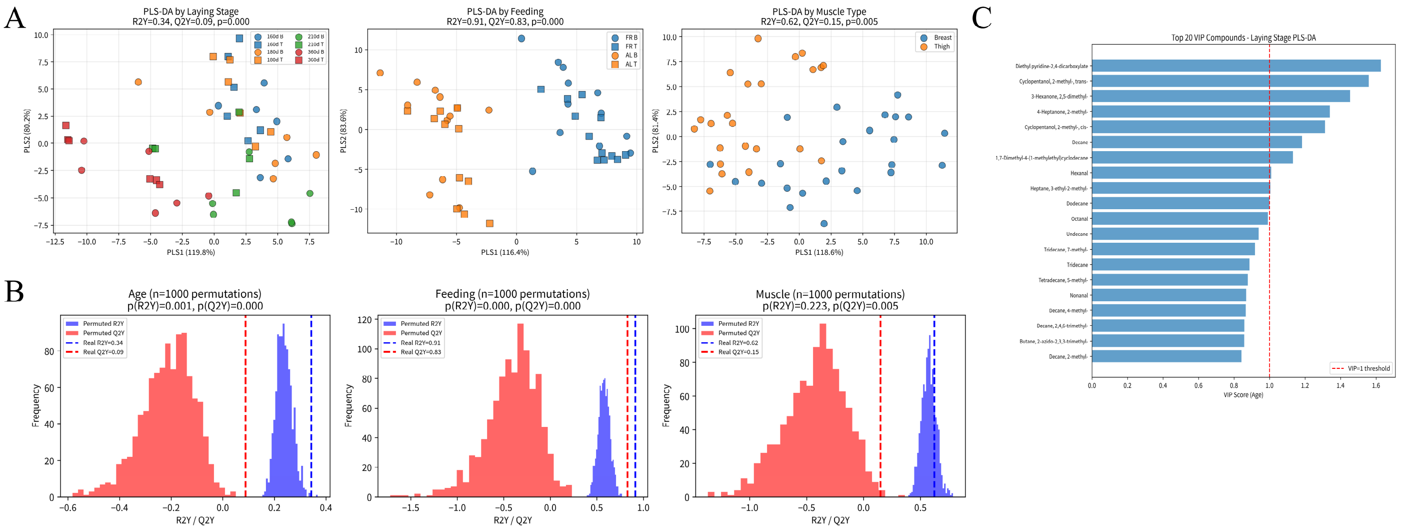

Supplementary Figure S1. Multivariate analysis of GC-MS volatile profiles. (**A**) PLS-DA score plots colored by laying stage, feeding regime, and muscle type (CLR-transformed data, 47 samples × 79 compounds). (**B**) Permutation test results (n = 1000) for each PLS-DA model, showing observed R2Y/Q2Y (dashed lines) against permuted distributions. (**C**) VIP score bar plot for the top 20 compounds contributing to laying-stage discrimination.
